# The Influence of Reaming Velocity During Preparation of the Femoral Canal—An In Vitro Analysis of Two Straight Femoral Revision Stems with a Fluted Tapered Design

**DOI:** 10.3390/bioengineering12090984

**Published:** 2025-09-16

**Authors:** Oliver E. Bischel, Jörn B. Seeger, Matthias K. Jung, Stefan Dörfler, Arnold J. Höppchen, Alexander Jahnke, Eike Jakubowitz

**Affiliations:** 1 BG Trauma Center, University of Heidelberg, Ludwig-Guttmann-Str. 13, 67071 Ludwigshafen, Germany; matthias.jung@bgu-ludwigshafen.de; 2 Kurpark-Klinik, Kurstr. 41-45, 61231 Bad Nauheim, Germany; joernseeger@gmx.net; 3 Laboratory of Biomechanics, Justus-Liebig-University Giessen, Klinikstr. 29, 35392 Giessen, Germany; active96@web.de (S.D.); alexander.jahnke@ortho.med.uni-giessen.de (A.J.); 4 Neckar-Odenwald-Hospital Mosbach, Academic Teaching Hospital of Heidelberg University, Knopfweg 1, 74821 Mosbach, Germany; arnold.hoeppchen@neckar-odenwald-kliniken.de; 5 Department of Orthopaedic Surgery, Medical Faculty Mannheim of Heidelberg University, Theodor-Kutzer-Ufer 1-3, 68167 Mannheim, Germany; 6 Laboratory for Biomechanics and Biomaterials, Department of Orthopaedic Surgery, Hannover Medical School, Anna-von-Borries-Str. 1-7, 30625 Hannover, Germany; jakubowitz.eike@mh-hannover.de

**Keywords:** modular revision stem, monobloc revision stem, revision THA, implant fixation

## Abstract

*Background:* The use of tapered fluted revision stems has been shown to be reliable and safe. Primary stability is mandatory for a long-lasting fixation between bone and a prosthesis. Nevertheless, aseptic loosening due to insufficient primary stability occurs and may be related to technically improper preparation of the femoral canal. Instructions of manufacturers are heterogeneous regarding preparation of implant beds. *Questions/Purposes:* Does speed or the design of the reamer influence the accuracy of the implant bed and, consecutively, primary stability? *Materials and Methods:* A test foam with an elastic moduli and pressure resistance similar to that of cancellous bone was used. The medullary canal was prepared with the use of reamers of two different straight and tapered femoral revision devices. Three different rotational speeds were used for preparation. After preparation, primary stability was measured and fixating characteristics were derived. *Results:* Sufficient primary stability was achievable by all three preparation methods but fixating characteristics were different. Significantly higher micro-motions were detected near the tip of the prosthesis compared to those at all more proximal measuring points. Reaming with high velocity resulted in significantly higher micro-motions compared to that with mid- or low-speed burring. *Conclusions:* Different preparation methods may be one explanation for the range of reported survivorship data of the two devices with aseptic loosening as the end point. The precision of the implant bed and fixating characteristics were best after reaming with lower velocity. Superior but not significantly better fixation characteristics were achieved with the monobloc stem compared to those with the modular device.

## 1. Introduction

Revision monobloc devices with a fluted and tapered design have undoubtedly improved outcomes after femoral revision total hip arthroplasty (RTHA). Since their introduction more than 30 years ago, modular devices with a straight or curved stem based on this fixation principle have been launched and mid-term results have also been promising [1,2]. Despite these encouraging results, aseptic loosening is still one of the main failure reasons.

Obtaining primary stability is mandatory for a durable fixation due to osseointegration. Compared to primary total hip arthroplasty (PTHA), the amount of bone defect is one factor influencing primary stability in RTHA. In addition, preparation of the femoral canal in cases of revision is more difficult and precise adaptation of the implant bed between the stem and (endosteal) bone is sometimes challenging.

Manufacturers’ guidelines are not standardized and reaming by hand or machine is individually performed. The aim of this study was to investigate influences on the quality of implant bed preparation resulting from technical adjustments like speed and the feed of the reamer. We analyzed two different tapered and fluted revision stem devices and their corresponding reamers, one monobloc stem (Wagner SL^®^, Zimmer Biomet, Warsaw, IN, USA) and one straight modular system (Link MP^®^, Waldemar Link GmbH & Co. KG, Hamburg, Germany).

## 2. Materials and Methods

**Figure 1:**
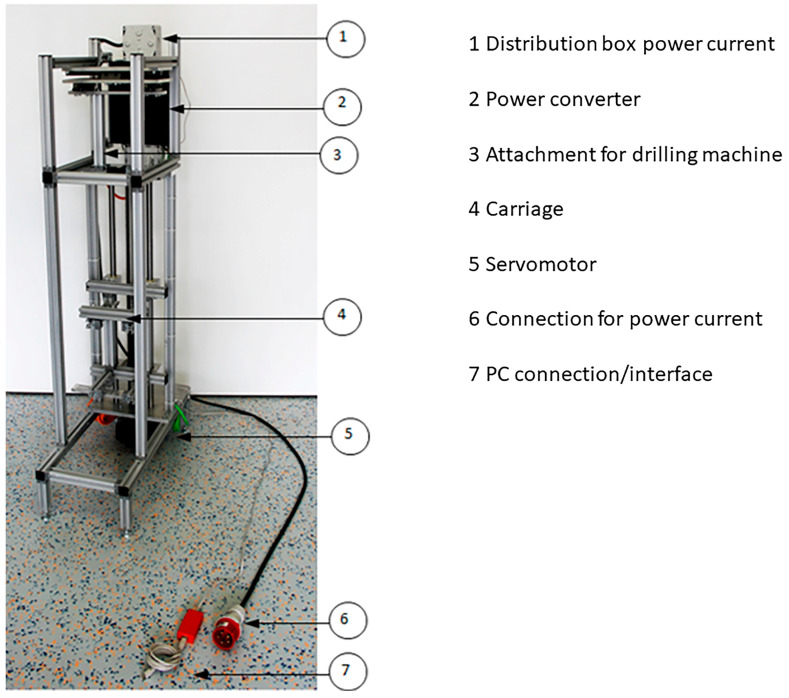
Principal breadboard.

A commercial hand drilling machine (BE 1100, Vario-Tacho-Constamatic, Metabo, Nürtingen, Germany) was integrated at the top of a custom-made test setup similar to a box column drill (Figure 1).

**Figure 2:**
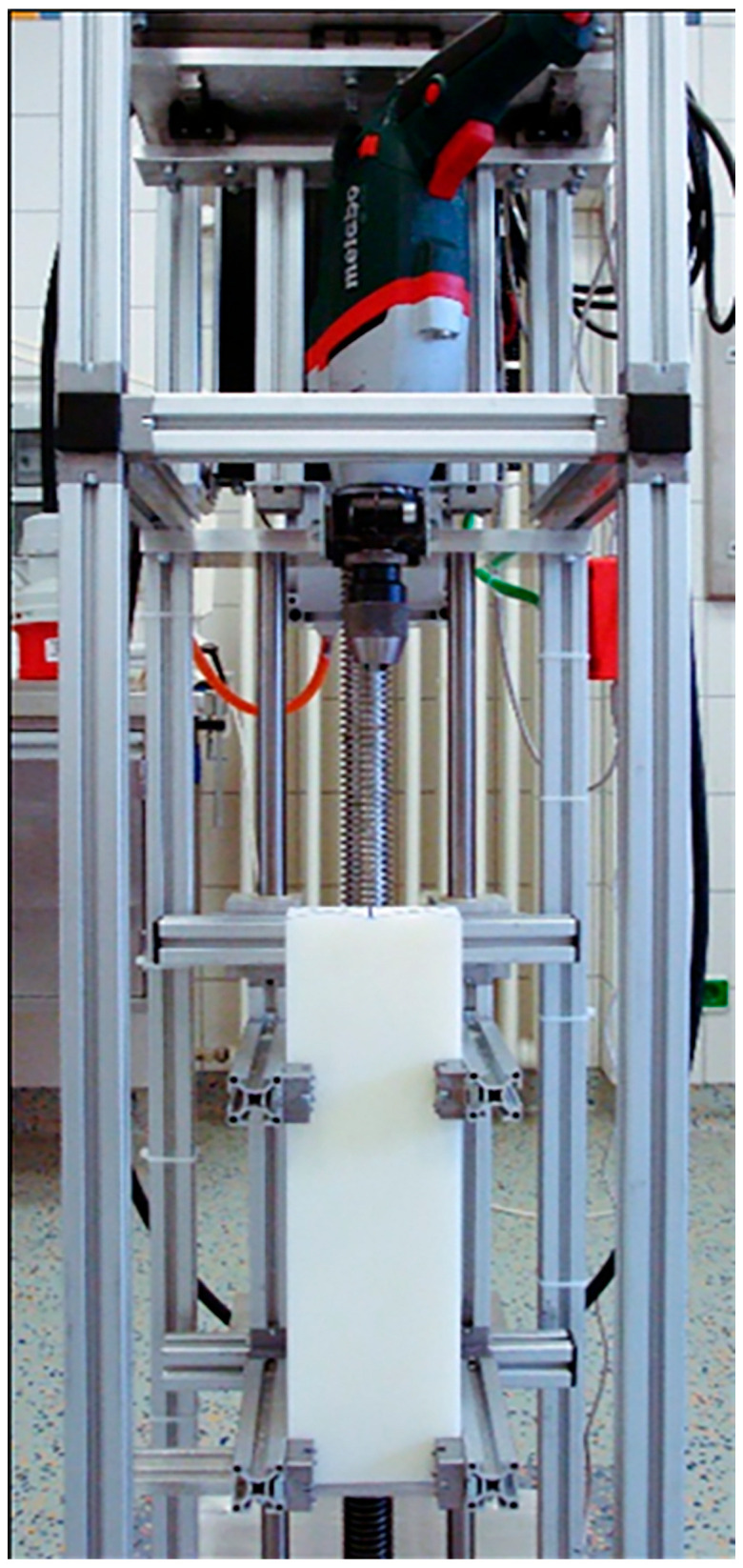
Drilling machine placed perpendicularly to the specimen.

The drilling machine was integrated into the setup perpendicularly to the test specimens and coupled with an X-Y transverse force equalizer with two pairs of linear bearings (Figure 2).

The test specimens were fixed in a holder and for a defined feed; this holder was moved against the drilling machine via a servomotor that drove a fine-thread spindle. Three different speeds were used to prepare the medullary canal, similar to those used in operating theatre (OR) drilling machines: 1000 rounds per minute (rpm) for the machined drilling mode, 250 rpm for the machined reaming mode, and 70 rpm representing manual reaming.

In biomechanical investigations, standardized bone models such as Sawbones are frequently used. These are typically equipped with polyurethane (PU) foam cores intended primarily for shaping the artificial cortex. However, such cores are considerably softer than human cancellous bone and lack any mechanical bonding to the cortical shell. In the biomechanical literature, higher-density PU foam has therefore been employed as a model for human cancellous bone [3]. For example, Zerdzicki et al. [4] recently demonstrated that PU exhibits compressive and tensile properties comparable to those of osteoporotic human trabecular bone. Horák et al. [5] even reported that PU foam is both mechanically and thermally stable and suitable for testing surgical instruments in drilling procedures.

**Figure 3:**
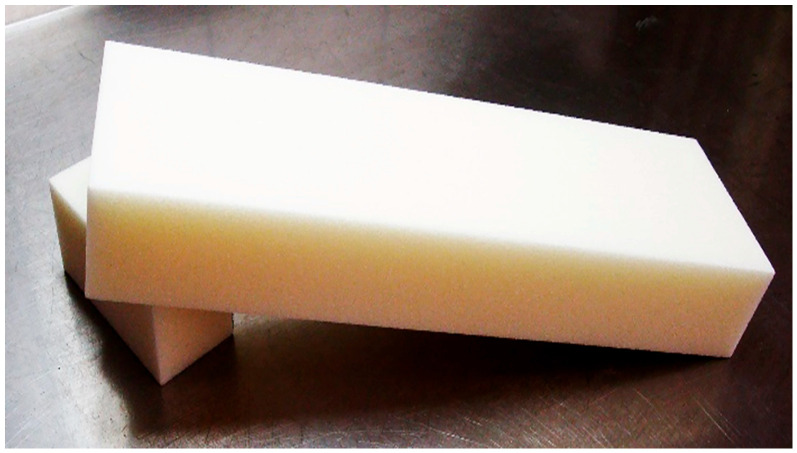
PMI foam (350 × 100 × 50 mm).

A closed-cell rigid Polymethacrylimid (PMI) foam with an elastic modulus of 350 MPa and a pressure resistance of 9 MPa was used as test blocks measuring 350 × 100 × 50 mm (Figure 3; Rohacell^®^ 200 WF, Evonik, Essen, Germany) [6]. This foam was used since the elastic modulus and pressure resistances were comparable to those of the cancellous bone of the proximal and distal femur [7,8].

Two established stem devices with a tapered body and sharp ridges to gain rotational stability were selected for this study (Wagner SL^®^ and Link MP^®^). Both implant systems are made of titanium alloy (Wagner SL^®^: Protasul 100^®^, Ti-6Al-7Nb; Link MP^®^: Tilastan^®^, Ti-6Al-4V) for cementless use and developed as revision devices for diaphyseal anchoring to bridge bony defects of the proximal femur. The Wagner SL^®^ stem, as a monobloc implant, shows a tapered distal anchoring area with sharp ridges to prevent rotational stability with a neck angle of 135 degrees. The Link MP^®^ prosthesis is modularly built-up with a tapered and fluted distal body and two neck options, each in length (35 and 65 mm) and neck angle (135 and 126 degrees). Indications for both devices are identical. The chosen diameter of both devices was 20 mm. The SL stem had a cone angle of 2° and a length of 265 mm. The MP stem was 250 mm long with an angle of 3°. The corresponding reamers of both systems were used for preparation of the implant bed. The reamers were conically formed but showed significant differences. The preparation tool of the SL system was designed similarly to a drill with non-sharpened cutting edges arranged helically around the longitudinal axis. The sharpened rips of the MP reamers were orientated in a straight line parallel to the longitudinal axis. According to the surgery guidelines of the systems, the implant bed was subsequently prepared stepwise with corresponding reamers after pre-drilling the foam blocks with commercial drills with 10 and 16 mm diameters. This pre-drilling was performed in a standardized manner using a feed of 2 mm/s and a speed of 100 rpm. To prevent thermal effects, constant cooling with water was ensured. Ascending reaming for both RTHA systems was performed starting with an 18 mm drill and up to the final diameter of 20 mm.

A total of four foam blocks were prepared for each drilling speed, resulting in a total of 24 blocks.

The outer shapes of the foam blocks were cut to create cylindrical specimens, resulting in equal material wall thickness with respect to the drilled holes. The stem of each RTHA system was then inserted by hand and impacted by a materials testing machine (Type: Inspect Table Blue, Hegewald & Peschke, Nossen, Germany) using 25 cycles at first with an impaction force of 2 kN (representing surgical impaction) and then 25 cycles with 4 kN (representing patient loading) to generate a standard press-fit fixation [9,10]. Four relative micro-motions, rm1-rm4, were tracked as a function of four measuring points with equal distributed distances placed on each foam cylinder (B1-B4) and RTHA stem (P1–P4) by applying an axial moment, simulating parts of the rotational component of activities of daily living for the stem. An established measuring setup using tactile transducers was used [11]. During the post-processing, differences in micro-motions regarding measuring point pairs at the same level on the stems and the foam cylinders were calculated, resulting in relative micro-motions. The abscissa of motion graphs represents the level of captured motions as a function of the measuring point, whereas the ordinate stands for normalized motion in mdeg/Nm. These were analyzed and compared between both RTHA systems.

SPSS (Version 22.0, IBM Corp., Armonk, NY, USA) was used for statistical calculations. Analysis of variance (ANOVA) with a post hoc least significant difference (LSD) test was performed to compare the relative micro-motions within each single group, to characterize the specific stem fixation and to compare different groups. A value of *p* ≤ 0.05 was considered to be significant. In the event of an insufficient number of cases, a post hoc case number analysis was carried out using the data collected here and the G-Power system.

## 3. Results

### 3.1. Link MP^®^ Reconstruction Stem

**Figure 4:**
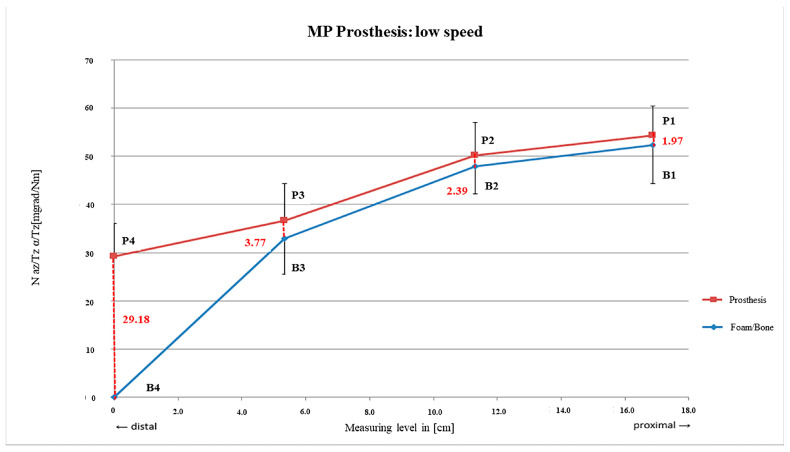
MP prosthesis preparation with slow rpm.

**Figure 5:**
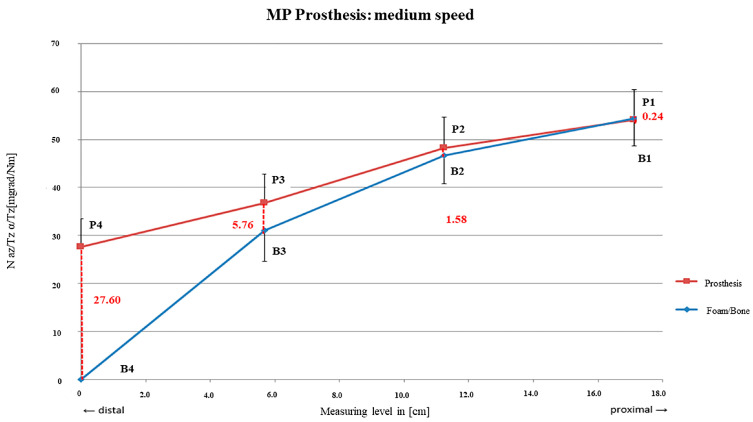
MP prosthesis preparation with medium rotational speed.

**Figure 6:**
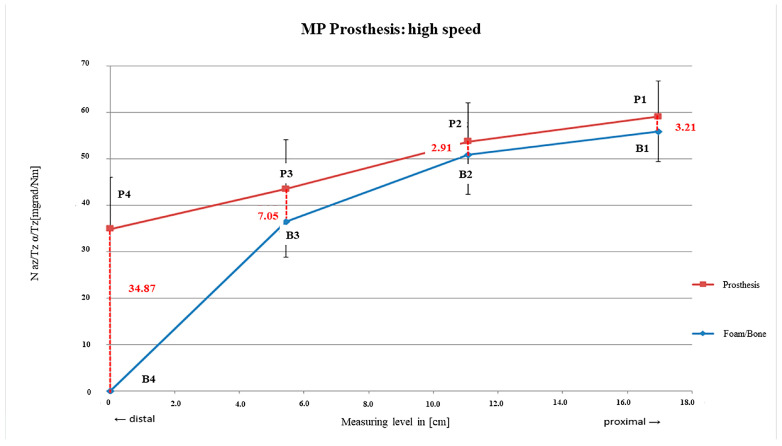
MP prosthesis preparation with high-speed burring.

As a function of the drilling speed, the MP system showed almost parallel curves of the prosthetic and bony motions with increasing micro-motions from proximal to distal assuming a fixation pattern of a more proximal fit decreasing to the tip after preparation at all velocities (Figure 4, Figure 5 and Figure 6). Primary stability was achieved with all three drilling speeds.

Nevertheless, preparation with low speed resulted into the narrowest distance between the two curves on average (rm_1_ = 1.97 mdg/Nm; SD = ±2.08 mdg/Nm) with an almost constant and parallel deviation between rm_2_ and rm_3_ until rm_4_ related to increased micro-motions (rm_4_ = 29.18 ± 6.90 mdg/Nm). Consecutively, the main fixation was located between rm_1_ and rm_3_ and, thus, along more than the proximal two thirds of the stem (Figure 4).

Preparation with mid-speed resulted in more steeply decreasing curves since the proximal rm1 area showed lower relative motions (rm_1_ = 0.24 mdg/Nm; SD = ± 0.62 mdg/Nm) than rm_2_ and rm_3_ and, thus, a shorter main fixation area (Figure 5).

Even after preparation at high speed, the curves between rm_1_ and rm_2_ were almost parallel and again comparable to those of the slowly drilled foam. The smallest relative motions within the main fixation area were in the upper third of the stem with rm_2_ = 2.91 mdg/Nm; SD = ± 1.35 mdg/Nm. In the rm_3_ area, the largest relative motions were suddenly observed in the actual main anchoring area compared with the other two drilling speeds (Figure 6).

### 3.2. Wagner SL^®^ Revision Stem

**Figure 7:**
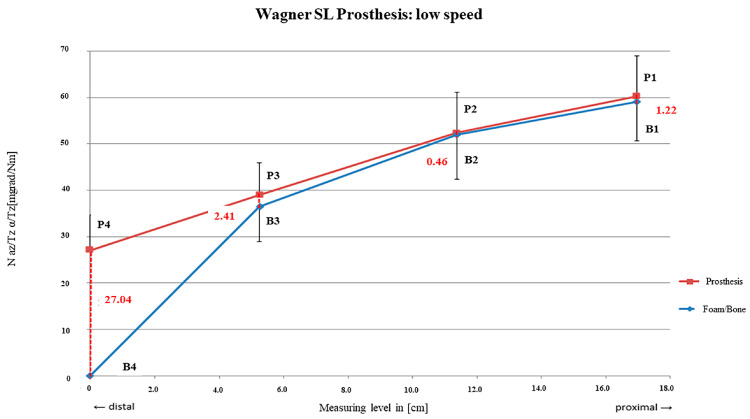
SL prosthesis preparation with slow rpm.

**Figure 8:**
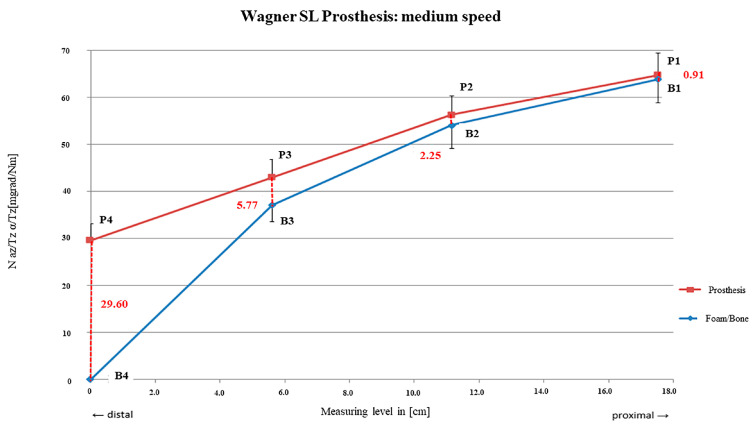
SL prosthesis preparation with medium rotational speed.

**Figure 9:**
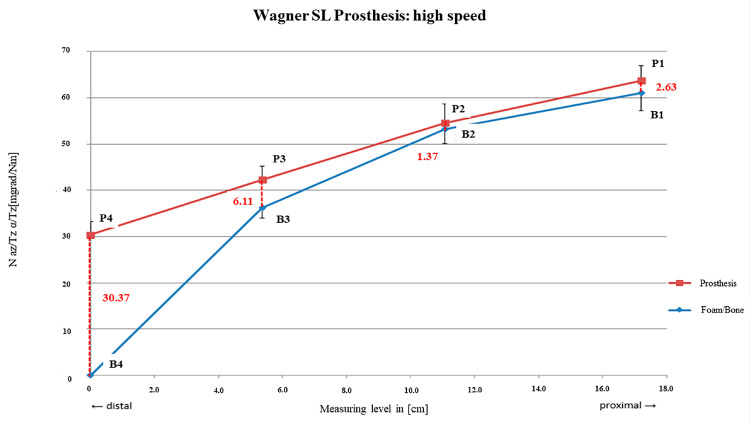
SL prosthesis preparation with high-speed burring.

Similarly to those of the MP prosthesis, both curves showed an almost parallel progression, revealing a proximal fixation distance over two thirds of the stem. Sufficient primary stability was achieved by all three preparation procedures (Figure 7, Figure 8 and Figure 9).

The distance between the curves was relatively small between rm_1_ and rm_3_, with rm_2_ = 0.46 mdg/Nm (SD = ± 0.95 mdeg/Nm) showing the tightest fixation area. The two curves also diverged from rm_3_ towards rm_4_, representing a decreasing contact area more distally until the tip of the stem (rm_4_ = 27.04 mdg/Nm; SD = ± 7.60 mdg/Nm; Figure 7).

A continuous increase in the distance of the two curves from proximal to distal was present after preparation with mid-speed. Relative motions were higher than after preparation with low speed with rm_1_ = 0.91 mdg/Nm (SD = ± 0.67 mdg/Nm) and rm_4_ = 29.60 mdg/Nm (SD = ± 3.56 mdg/Nm). However, the main fixation area was also present between rm_1_ and rm_3_ (Figure 8).

Higher micro-motion was measured after high-speed burring compared to preparation with low speed and mid-speed. With rm_2_ = 1.37 mdg/Nm (SD = ± 1.02 mdg/Nm), the main fixation area was detected with lowest relative motions between the foam and stem. However, a relatively close gradient was visible towards rm_1_ and rm_3_, also limiting the main fixation area for the proximal two thirds of the stem. A steep increase was also obvious for rm_4_ = 30.37 mdg/Nm; SD = ± 2.86 mdg/Nm (Figure 9).

### 3.3. Comparison of the Two Systems

Both systems showed significant differences with significantly lower relative micro-motions between the stem and foam for the SL device (*p* < 0.01). Significant differences could also be detected for both the measuring levels (*p* < 0.01) as well as between the reaming speeds used for the preparation (*p* < 0.01). The distal relative motion rm_4_ always showed significantly higher values compared to all other relative motions, rm_1_-rm_3_, independently of the stem used (*p* < 0.01). In contrast, rm_1_-rm_3_ for both investigated stems were always comparable (*p* > 0.05). Regarding the reaming speed, there was a significant difference in stem micro-motions between preparations at high (1000 1/min) and low speeds (70 1/min; *p* < 0.01) as well as between those at high speed and mid-speed (250 1/min; *p* = 0.01). With regard to fixation characteristics, both systems did not differ despite a tendency towards the tighter fixation of the SL device (*p* > 0.05).

### 3.4. Post Hoc Power Analysis

A post hoc sample size calculation based on the present data with alpha (5%) and beta (20%) errors (power: 80%) and the mean difference between relative movements of the fastest and the lowest reaming speeds (∆rm = 7.27 mdg/Nm; SD = ± 7.60 mdg/Nm) resulted in a necessary sample size of *n* = 19 per reaming group in order to gain significant differences.

## 4. Discussion

### 4.1. Link MP^®^ Reconstruction Stem

The lowest relative micro-motion of 8.94 mdeg/Nm on average from proximal to distal was detected with slowest reaming velocity in this setup. As the curves were nearly parallel, a continuous fixation could be derived from proximal to distal over the whole stem distance. Thus, a homogeneous loading of the bone along the conical conjunction of the stem should be provided and highest force transmission correlates with the contact surface from proximal to distal [12].

Higher micro-motions of 9.54 mdeg/Nm on average were seen when reaming with mid-speed with highest relative motions distally decreasing to proximal. The fixation range was therefore shorter and predominantly in the proximal stem area which is crucial to achieve primary stability [13,14,15].

Even higher relative micro-motions were detected using highest drilling speed with 11.38 mdeg/Nm on average. The fixation characteristics were similar to those with lowest drilling speed but may have occurred more distally. This may also be the fact in revision cases with a proximally compromised bone situation due to persisting defects or bone loss in the fixation area of the former stem due to mechanical failure. In summary, using lower velocities during reaming may result in decreased micro-motions and a longer fixation area as the femoral bed may be prepared more precisely. Preparation of the femoral bed may be one influencing factor of the range of implant survival after five years with aseptic loosening as the end point, which is between 92.5 and 99% [15,16,17,18,19,20,21].

### 4.2. Wagner SL^®^ Revision Stem

An average relative micro-motion of 8.18 mdeg/Nm from proximal to distal subsuming all measuring points was detected after preparation with lowest drilling speed with a short main fixation area located near the proximal third of the stem (at rm_2_). This finding was similar to that of a former analysis of Jakubowitz et al. with the same implant [11].

A slight but insignificant increase in the average micro-motion to 8.87 mdeg/Nm and mainly proximal anchoring characteristics were present after preparation with the mid-reaming speed decreasing continuously from proximal to distal.

The highest relative micro-motion of 10.60 mdeg/Nm was seen after preparation with high-speed burring. Anchoring characteristics with a main fixation area around measuring points P2/B3 were similar to those after low-speed burring but over a longer distance.

### 4.3. Comparison of Both Systems

The closer the lines of the micro-motions detected at the measuring points of the stems and foam cylinders ran to each other, the higher the primary stability was. Overall relative micro-motions of 100–150 µm or more prevent bony integration [22,23]. Osteointegration may be safely achievable with motions lower than 40 µm [15] but even higher micro-motions may be present in vivo and have to be taken into account [24]. Therefore, presupposition for bony integration and long-term duration may be achievable with both systems and all revelation speeds used within this setup. In addition, similar anchoring characteristics of both devices were seen with the different revelation speeds although significantly higher micro-motions were detectable after high-speed burring compared to mid- or low-velocity preparation. The Wagner SL device showed advantageous characteristics compared to the MP system. Influencing factors may be design-related with regard to the reamer characteristics, such as whether it is sharpened or not or whether it has straight or spindle-like ridges, and the stem design itself.

Lower revelation speed may be advantageous with respect to implant fixation. Higher pressure may be necessary to obtain feed and to prepare the implant bed with lower revelation speeds, especially when reaming by hand. The risk of induction of a periprosthetic fracture may be higher in patients with lower speed during reaming and also when inserting the implant [16].

Although the taper design of both devices is able to gain enough primary stability more distally underneath potential bone defects [11], a certain amount of contact area between bone and prosthesis is necessary for primary stability [13,25,26]. A contact area of at least 40% is thought to be sufficient [15]. An anchoring area around two measuring points was achieved with both implants, corresponding approximately to 40–50% of the contact area. Even with the Wagner SL device and preparation with low velocity, a contact area of at least 30% was detectable and seems to be sufficient with respect to clinical data and survival of over 90% after 14 years with aseptic loosening as the end point [27].

### 4.4. Limitations

Although a post hoc power analysis indicated that a sample size of 19 per group would be needed to reach statistical significance, practical constraints—namely the high complexity and time demand of our drilling and measurement protocol—make such a number infeasible under normal laboratory conditions. Nevertheless, the observed trends were consistent across all samples, with effect sizes suggesting a likely influence of drilling speed on primary stability. We therefore interpret our study as explorative, offering valuable directional insight that can guide future confirmatory research.

The structure of human bone is influenced by several factors like the origin of the individuum, sex, age, and activity of daily life. Therefore, a high variety of physical parameters of human bone are reported and suitable material as a substitute for human cancellous bone is difficult to find. Consecutively, results obtained from investigations using synthetic bone substitutes should be critically checked and transferability to clinical routine may be limited.

Heat dissipation during reaming with higher revolution was present despite constant cooling with water, although deformation of the polymethacrylamide blocks could not be seen macroscopically. As heat exposure was not measured in this investigation, damage in any way could not be ultimately excluded. The use of thermocouples may be one option to quantify the effectiveness of cooling during preparation in further investigations. Although the elastic modulus and compression strength were adapted to the cancellous bone of the proximal femur, reaction to frictional heat may be different in situ as blood and components of bone marrow may lead to local cooling. An increase in temperature up to 62 degrees Celsius for a duration longer than two seconds may result in persisting damage to bone and surrounding tissue. In this context, irrigation of the femur is mandatory to reduce intramedullary pressure [28]. Further investigations may be necessary, dealing with the microanatomic response of the bone with different preparation options.

A concentricity of 100% was not achievable with the used reamers and revolution speeds but concentricity should be lesser in vivo as left cement or sclerotic bone may lead to excentric reaming. Clinically, reaming using high speed and the use of the reamer as a mill is one option to bypass those sclerotic areas. Otherwise, reaming using low revolution speeds may be more accurate in cancellous bone. In addition, cortical bone was not considered in this setup. Unilateral contact of the reamer with cortical bone may also lead to excentric reaming when a straight reamer is used in a bowed femoral bone. The anchoring area should be limited due to this bowing. Two bone diameters for stem fixation are sufficient when a circumferential endosteal bone to implant contact area can be achieved. Thereby, the influence of excentric reaming is limited.

The reamers of both systems were different. Non-sharpened, fluted, and spindle-like reaming ridges were used with Wagner SL whereas the MP system had sharpened and straight ridges. Nevertheless, there was no detectable significant influence in this study.

## 5. Conclusions

This in vitro investigation revealed better results with low revelation speeds used for preparation of the implant bed. The best results were achieved with the Wagner SL device with respect to the fixation characteristics and primary stability but were not significant compared to those of the MP revision device. Concentricity was inferior with low velocities and may also have had a positive influence during preparation of the implant bed. Nevertheless, preparation may also be influenced by bone defects, missing cancellous bone, sclerotic bone, or even left PMMA particles. Due to these factors, transferability to clinical practice may be limited.

## Data Availability

The data presented in this study are available on request from the corresponding author. The data are not publicly available due to privacy issues.
